# Assessment of Photodegradation and Biodegradation of RPU/PIR Foams Modified by Natural Compounds of Plant Origin

**DOI:** 10.3390/polym12010033

**Published:** 2019-12-24

**Authors:** Joanna Liszkowska, Marcin Borowicz, Joanna Paciorek-Sadowska, Marek Isbrandt, Bogusław Czupryński, Krzysztof Moraczewski

**Affiliations:** 1 Department of Chemistry and Technology of Polyurethanes, Institute of Materials Engineering, Kazimierz Wielki University, J.K. Chodkiewicza Street 30, 85-064 Bydgoszcz, Poland; m.borowicz@ukw.edu.pl (M.B.); sadowska@ukw.edu.pl (J.P.-S.); m.isbrandt@ukw.edu.pl (M.I.); czupr@ukw.edu.pl (B.C.); 2 Department of Polymer Materials Engineering, Institute of Materials Engineering, Kazimierz Wielki University, J. K. Chodkiewicza Street 30, 85-064 Bydgoszcz, Poland; kmm@ukw.edu.pl

**Keywords:** rigid RPU/PIR foam, DSC, FTIR, thermal degradation, biodegradation, climatic chamber, cinnamon extract, coffee extract, cocoa extract

## Abstract

Four types of rigid polyurethane-polyisocyanurate foams (RPU/PIR) were obtained. Three of them were modified by powder fillers, such as cinnamon extract (C10 foam), green coffe extract (KZ10), and cocoa extract (EK10) in an amount of 10 wt %. The last foam was obtained without a filler (W foam). The basic properties and thermal properties of obtained foams were examined. All foams were subjected to degradation in the climatic chamber acting on samples of foams in a defined temperature, humidity, and UV radiation for 7, 14, and 21 days. The physico-mechanical properties of foams were tested. The compressive strength of degraded foams after 7, 14, and 21 days was compared with the compressive strength of nondegraded foams (0 days). The chosen properties of degraded foams, such as cellular structure by scanning electron microscopy (SEM) and changes of chemical structure by FTIR spectroscopy were compared. The obtained foams were also subjected to degradation in a circulating air dryer in an increased temperature (120 °C) for 48 h. Additionally, W, C10, ZK10, EK10 foams were placed in a soil environment and subjected to 28 days biodegradation process. The biochemical oxygen demand (BOD), the theoretical oxygen demand (TOD), and the degree of biodegradation (*D*_t_) of foams were determined in this measurment. Test results showed that the compressive strength of foams decreased with the longer time of foam degradation in the conditioner. The foam subjected to degradation darkened and became more red and yellow in color. The addition of natural compounds of plant origin to foams increased their susceptibility to biodegradation.

## 1. Introduction

Polyurethanes (PU) have become widely used polymers in recent years. Foams are particularly widespread. They can be found in sports and recreational products, in military applications, in automotive industry, in airplanes, in furniture, in packaging, in the insulation, in toys, etc. [1,2,3]. They are also used as a protection during the transport of goods [4,5]. The production of polyurethane foams (PUF) is still dependent on petroleum [6]. The concerns relate primarily to the harmful health effects and environmental problems of oil-based PU and future crude oil shortage [7]. The development of new technologies motivates searching for solutions based on natural and renewable raw materials with such chemical structure that it will allow their quick and easy degradation [4,5,6,7,8]. The need to develop a more ecological polyurethane material from renewable sources is also associated with an increasing amount of waste from PU. The polyurethane foams market is increasing day by day. The amount of waste generated from the production process and from post-consumer products also increases at a very high rate [8,9]. Therefore, polyols from renewable sources are an obvious alternative to polyols based on petroleum. They have an influence on increase biodegradability of polyurethanes. Thus, we can directly influence on the decrease of PU waste amount using these raw materials. Therefore, current research is being conducted into bio-polyols, which have a structure susceptible to degradation. They can be obtained, e.g., from biomass residues, vegetable oils, or industrial by-products [7,10,11,12,13,14]. Seed oils are the most promising and most intensively tested in the PU industry among natural resources. This is primarily due to their high availability, low price, and biodegradability [6]. PU from seed oil-based polyols are more susceptible to microbial degradation than those from fossil resources. Biodegradability of this material depends on the presence of certain chemical groups in the molecule. Easily hydrolyzing amide or urethane groups accelerate polyurethane degradation [15]. Ester groups that occur naturally in the structure of seed oils are also easily biodegradable [16,17,18]. 

Starch is one of the natural compounds, which is used to increase the biodegradability of polyurethanes. Lubczak and Szczęch obtained a polyol compound that caused biodegradation of rigid foams [19]. They dissolved starch in formalin (36%) and heated it at 80–85 °C. O-hydroxymethyl derivatives were obtained. In the next step, the soluble O-hydroxymethyl derivative of starch was used for reaction with glycidol to obtain a semi-product (bio-polyol) for production of polyurethane foam. The effect of the main components of the formulation, used to make polyurethane foams (PUF) derived from unrefined crude glycerol (CG), was investigated in an effort to develop environmentally friendly materials [20]. Sousa et al. and Veronese et al. studied the production of bio-foams for energy absorption materials from castor oil and cellulose microfibers in reaction with 1,6-hexamethylene diisocyanate (HDI) [21,22]. The ground waste of plant origin, such as rapeseed cake [23], sugar beet pulp [24], cork [25,26], chitosan [27], corn starch [28], corn bran [29], corn stover and rice strav [30], corn stalk [31], corncob [32], lignin [33,34], bark [35,36,37,38], wheat straw [39], cotton stalks [40], apple pomace [41], or linseed cake [42] were used as physical fillers for the production of RPU/PIR bio-foams.

Polymer degradation may occur during their aging. This process may include: A physical aging (without chemical reaction); a chemical change (cross-linking during thermoset curing); thermal conditioning at elevated temperatures or photochemical aging [42,43,44]. Aging of materials is the structural change occurring in the whole mass of the material for a long time [45]. Accelerated aging is the process of a simulation of natural aging in a shortened time. Aging of polyurethane foams is tested mainly by measuring changes of linear dimension, geometric volume, and mass after 48 h of thermostating in a dryer with forced circulation [46]. Research shows that the aging properties of foams based on renewable raw materials are worse than properties of foams based on petroleum raw materials. Thus, a lot of studies are carried out to improve the mechanical, thermal properties, and flammability of foams by introducing various fillers and flame-retardant additives. Structural changes in the polymer may occur during processing, storage, or exploitation. These changes cause the loss of original properties. The first visible symptom of this phenomenon is color change (yellowing) and tarnishing of polymer surfaces. Chemical (e.g., stabilizers, antioxidants, UV absorbers) and physical modification are used to prevent this [47]. These additional components in the material can react at elevated temperatures with the polymer [48]. UV radiation contained in sunlight is richer in energy than visible light. It causes photochemical degradation of polymers. UV absorbers are used to protect the plastic. The materials without stabilizers become brittle, discolored, or cracked as a result of solar radiation. UV absorbers absorb harmful ultraviolet radiation in the wavelength range of 290–400 nm. They turn light energy into harmless heat.

Addition of antioxidant compounds is necessery to protect foams against damage by UV radiation, for example during external use. It would be good if the same compounds simultaneously caused the degradation of foams in properly created conditions (e.g., when mixed with soil or with compost). They are used to protect plastics against photo- or thermooxidation by neutralizing free radicals and peroxides resulting from the decomposition of e.g., polyolefins. They can be phenol, trivalent phosphorus, or thioesters derivatives. Currently, chemical stabilizers are offered on the market, which in some conditions may not necessarily have a good influence on human health (e.g., UV stabilizers based on benzophenone, benzotriazole, triazine, or amine) [49].

Therefore, it is justified to use stabilizers of natural origin in the form of powdered extracts of green coffee (KZ), cocoa (EK), or cinnamon (C). Each antioxidant should meet the following requirements—be in a solid state to limit migration [49], be miscible with polymers, have high thermal stability, be nontoxic (especially when it is used in food packaging), and be relatively inexpensive. The migration of harmful chemical compound rules is set out in applicable national and international law [50,51]. All these requirements are met in the case of three extracts used in this article (KZ, EK, C). They contain effective plant antioxidants such as: Chlorogenic acids, phenolic acids, polyphenols, and alkaloids.

The main aim of this research was to check the effectiveness of plant-based antioxidants, such as extracts of green coffee, cocoa, and cinnamon, on the degradation and biodegradation process of RPU/PIR foams. Three methods of the degradation process of RPU/PIR foams were used to this research: Accelerated aging in the air conditioner with simultaneous acting of UV radiation, temperature and humidity; resistance to long exposure to high temperature in the dryer with forced circulation of air; resistance to soil environment.

## 2. Materials and Methods

### 2.1. Materials

Rokopol RF-551—sorbitol oxyalkylation product (hydroxyl number—420 mg KOH/g, molecular weight—650 g/mol, functionality—4.5, produced by PCC Rokita S.A., Brzeg Dolny, Poland) was used as a reference polyol, to prepare RPU/PIR foams. The catalytic system in RPU/PIR formulation were 33% solution of anhydrous potassium acetate (produced by Chempur, Piekary Slaskie, Poland) in diethylene glycol (produced by Chempur, Poland), as a trimerization catalyst and 33% solution of DABCO (1.4-diazabicyclo[2.2.2]octane, produced by Alfa Aesar, Haverhill, MA, USA) in diethylene glycol, as a polyurethane bond catalyst. The stabilizer of foam structure was poly(oxyalkilene siloxane) surfactant Tegostab 8460 (produced by Evonik, Essen, Germany). Carbon dioxide produced in situ in the reaction between water and isocyanate groups was a blowing agent. Furthermore, a commercial flame retardant Antiblaze TCMP—tris-(2-chloropropyl) phosphate (produced by Albemarle, Charlotte, NC, USA) was added into some of the foams. The isocyanate raw material was a technical polymeric diisocyanate Purocyn B (supplied by Purinova, Bydgoszcz, Poland), whose main component was 4,4′-diphenyl-methane-diisocyanate (MDI). Density of Purocyn B at a temperature of 25 °C was 1.23 g/cm^3^, viscosity was 200 mPas, and content of –NCO groups was 31.0%. Polyether and diisocyanate were characterized in accordance with appropriate standards such as ASTM D 2849-69 and ASTM D 1638–70.

Three natural compounds of plant origin, such as coffee extract, cocoa extract, and cinnamon extract (produced by Agrema Sp. z o.o., Agrema Sp. z o.o., Wroclaw, Poland) were used (Table 1) to the synthesis of RPU/PIR foams. Coffee extract contained 45 wt % of polyphenols, while cocoa extracts and cinnamon contained 5 wt % of polyphenols according to the technical data sheets of these natural compounds [52]. The main polyphenols in coffee, cocoa, and cinnamon extracts were chlorogenic acid, flavonoids, and phenolic acids [53]. The rest of the extracts was an inert carrier of active substance (cellulose, lignin, polysaccharides) [4,54,55].

### 2.2. Synthesis of the Rigid PUR/PIR Foams

Foam formulations (Table 2) were calculated based on the reference sources [56,57]. Detailed calculations are provided in articles [58,59]. RPU/PIR foams were prepared in a laboratory scale by one-step method from the two-component system (A and B) in the chemical equivalent ratio (*R*) of NCO groups to OH groups equal 3.7:1 (isocyanate index—370) [46]. An excess of polyisocyanate (3.7 *R* instead of 3.0 *R*) was used for reaction polyisocyanate with water. The NCO group chemical equivalent (*R*) was calculated according to Equation (1):

(1) $$R_{NCO}=\frac{4200}{31\%\;\mathrm{NCO}}$$

where: % NCO—content of NCO group in polyisocyanate raw material (%).

The chemical equivalent of hydroxyl group (*R*) was calculated according to the equation:

(2) $$R_{OH}=\frac{56100}{\mathrm{HN}}$$

where: HN—hydroxyl number of Rokopol RF-551 (mg KOH/g).

The component A (polyol premix) was obtained by the precise mixing of the appropriate amounts of Rokopol RF-551 (66.80 g), trimerization catalyst (8.00 g), urethane bond catalyst (3.20 g), flame retardant (47.60 g), surfactant (5.40 g), chemical blowing agent—CO_2_ generated in situ (distilled water was used in the amount of 3.15 g), and bio-filler. Component B was polyisocyanate Purocyn B in the amount of 250.60 g. Both components A and B were mixed at a respective mass ratio by mechanical stirrer (1800 rpm, 10 s) and poured into an open cuboidal mould with internal dimensions of 190 mm × 190 mm × 230 mm. A reference foam (W_0) were obtained in this way. The reference foam was modified by addition of plant-based fillers in the amount of 10 wt %. The amount of fillers was calculated in relation to the sum of the masses of main raw materials (polyol and polyisocyanate). Foam formulations containing 10 wt % of cinnamon extract (C10), green coffee extract (KZ10), and cocoa extract (EK10) were obtained (Table 2).

### 2.3. Methods

#### 2.3.1. Analyzing of Foaming Process

The foaming process was analyzed by an electronic stopwatch in accordance with ASTM D7487 13e^1^ [58]. This measurment to determine the characteristic foaming times: Cream time—from the start of mixing components A and B until appearing fine bubbles; free rise time—from the start of mixing the components A and B until the foam stops expanding; string gel time—from the start of mixing the components A and B until long strings of tacky material can be pulled away from foam surface when the surface is touched by tongue depressor; tack free time—from the start of mixing components A and B until the foam surface can be touched by tongue depressor without sticking. The maximum reaction temperatures (*T*_max_) in the foams were measured during synthesis, using a thermometer placed in the center of the obtained RPU/PIR foams.

#### 2.3.2. Accelerated Aging Test

Accelerated aging tests of the RPU/PIR foams was carried out in a thermostating process of cubic specimens with a side length of 50 mm at a temperature of 120 °C during 48 h. The samples were thermostated in a dryer with forced air circulation. The result of this test was the change of linear dimensions (Δ*l*), change of geometrical volume (Δ*V*), and mass loss (Δ*m*). Values of these parameters were calculated in accordance with ISO 1923:1981 and PN-EN ISO 4590:2016-11 from Equations (3)–(5).

(3) $$\Delta l=\;\frac{l-l_{0}}{l_{0}}\cdot 100\%$$

where: *l*_0_—length of the sample before thermostating, according to the direction of foam free rise (mm), *l*—length of the sample after thermostating, according to the direction of foam rise (mm).

(4) $$\Delta V=\;\frac{V-V_{0}}{V_{0}}\cdot 100\%$$

where: *V*_0_—geometrical volume of the sample before thermostating (mm^3^), *V*—geometrical volume of the sample after thermostating (mm^3^).

(5) $$\Delta m=\;\frac{m_{0}-m}{m_{0}}\cdot 100\%$$

where: *m*_0_—mass of the sample before thermostating (g), *m*—mass of the sample after thermostating (g).

#### 2.3.3. Aging in a Climate Chamber

This test consisted of a controlled treatment of destructive factors, i.e., increased temperature, humidity, and UV radiation at the same time. The mechanical properties play a significant role during the using foams in civil engineering, especially in building applications [59]. Aging of foams caused by acting of heat, UV radiation, and moisture, was measured in a set time interval of seven days (this series of foams were marked as C10_7, KZ10_7, EK10_7), of 14 days (marked as C10_14, KZ10_14, EK10_14), and of 21 days (marked as C10_21, KZ10_21, EK10_21). It consisted of placing samples in a heated heating chamber up to 50 °C, 70% relative humidity and irradiance of 320.86 W/m^2^. The climate chamber (DYCOMETAL CCK, model CCK-40/300 NG, Es-tor L.T.D., Poznań, Poland) was used for aging tests with artificial visible and UV light. The chamber had eight fluorescent lamps (PHILIPS SUPER ACTINICA TL 60W/10-R ISL). The fluorescence wavelength range was from 350 to 400 nm [60]. The dimensions of the chamber were 0.572 m × 0.654 m. Heating was set at 4 °C/min for the first h according to IEC 60068-3-5 (for empty chamber). Then, the temperature was kept at the constant level for a certain time (7, 14, or 21 days). Samples were placed directly under the lamps, so that the sample–lamp distance was as small as possible. The radiation from the lamps fell at an angle of 90° to the surface of the samples. The heating was carried out in a continuous mode, without opening the chamber. This action was aimed at deteriorating the physico-mechanical properties of the tested samples [61]. This did not fully reflect the changes which occur during the natural aging process, but it was sufficient to quantify the decrease of coefficient of compressive strength variation (CV) and to assess the effect of degradation conditions on the chemical structure (FTIR), thermal properties (DSC), and mechanical strength. The samples were removed from the chamber after ending the heating (after 1, 2, and 3 weeks of degradation). The degraded area was evaluated for selected properties of ambient conditions. The obtained results of selected tests of aged foams and nonaged foams were compared. 

#### 2.3.4. Apparent Density

The apparent density of the obtained foams was determined as the ratio of foam weight to its geometrical volume, using cubic samples with side length of 50 mm in accordance with ISO 845:2006 standard.

#### 2.3.5. Compressive Strength and Compressive Strength Ratio

Compressive strength was measured using an Instron universal strength machine 5544 according to PN-93/C-89071 (ISO 844:2014). The compressive strength of foams before degradation and after degradation was tested. The aging resistance in relation to the coefficient of variation of compressive strength (CV) was calculated from Equation (6) [62,63]:

(6) $$CV=\frac{W_{x}}{W_{0}}\cdot 100\%$$

where: *W*_0_—compressive strength measured before foam degradation (kPa), *W_x_*—compressive strength measured after foam degradation (kPa): *x* = 7 or 14 or 21.

#### 2.3.6. Differential Scanning Calorimetry (DSC)

Changes occurring in foams under heating were checked by differential scanning calorimeter DSC Q200 (TA Instruments, New Castle, DE, USA) with a built-in Advanced Tzero technology. DSC analysis was conducted in the range from −50 to 400 °C in one-step heating, under nitrogen flow. The mass of the sample was in range of 2.9–3.1 mg.

#### 2.3.7. Foam Structure

The foam structure was determined by Hitachi TM 3000 SEM microscope with an EDS attachment (Hitachi High-Technologies Co., Tokyo, Japan). The samples were dusted with a gold layer. The studies were performed at the accelerating voltage of 10 kV, with the working distance of 10 mm and magnification—50×. The statistical analysis of cell sizes was carried out on the basis of obtained micrographs by using ImageJ software (LOCI, Madison, WI, USA). A specialized program for measuring cells in the SEM method allowed the measurement of foam cell width and length. The anisotropy coefficient was calculated based on the width and height of the cells from Equation (7):

(7) $$\mathrm{Anisotropy}\;\mathrm{coeficient}=\frac{\mathrm{high}\;\mathrm{cells}}{\mathrm{width}\;\mathrm{cells}}$$

#### 2.3.8. Chemical Structure

The chemical structure of the obtained foams was evaluated on the basis of infrared spectra obtained by using the Nicolet spectrometer iS10 FTIR spectrophotometer (Thermo Fisher Scientific, Waltham, MA, USA). The measurement was carried out in a spectrospcopic range from 4000 to 400 cm^−1^ and maximum resolution of capability <0.4 cm^−1^ with DTGS detector. 

#### 2.3.9. Measurement of Foams Color

A standard colorimetric observer (2°), Konica Minolta CR-410, with a D65 light source and calibration according to white pattern (Konica Minolta Sensing Americas Inc, 101Williams Drive Ramsey, NJ, USA). The device gives the average of three measurements *L*, *a*, *b*. The difference between the two colors in the space (∆*E*) was calculated according to Equation (8):

(8) $$\Delta E=\sqrt{{\left(\Delta L\right)}^{2}+{\left(\Delta a\right)}^{2}+{\left(\Delta b\right)}^{2}}$$

where: *L*—the vertical axis of the coordinate system defining the brightness; *a*—axis of the cooirdinate the amount of red (positive ‘’*a*’’ values), the amount of green (negative ‘’*a*’’ values), b-axis expressing the amount of yellow color (positive values) or blue (negative values) in color.

#### 2.3.10. Susceptibility on Biodegradation

The study on the biodegradation process of RPU/PIR foams was carried out using the OxiTop® Control S6 apparatus (WTW-Xylem, Rye Brook, NY, USA), which used a respirometric method for measuring the oxygen demand for aerobic biodegradation of polymeric materials in soil [64]. The test was carried out in accordance with ISO 17556:2012. The biodegradation environment was garden soil with a moisture content of 5% (ISO 11274), pH 6 (ISO 10390), grain diameter < 2 mm. The biochemical oxygen demand (BOD) was determined from Equation (9):

(9) $${\mathrm{BOD}}_{S}=\frac{{\mathrm{BOD}}_{x}\;-{\;\mathrm{BOD}}_{g}}{c}$$

where: S—number of measurement days; BODs—biochemical oxygen demand of the analyzed material during S days (mg/L); BODx—measurement result for the x sample (mg/L); BODg—measurement results for the soil (without foam) (mg/L); *c*—sample concentration in the tested system (mg/L).

The degree of polymer biodegradation was determined on the basis of Equation (10):

(10) $$D_{t}=\frac{{\mathrm{BOD}}_{S}}{\mathrm{TOD}}\;\cdot 100\%$$

where: *D*_t_—degree of polymer biodegradation (%); TOD—theoretical oxygen demand (mg/L).

The theoretical demand oxygen (TOD) was calculated from Equation (11):

(11) $$\mathrm{TOD}=\frac{16\left[2c\;+\;0.5\left(h\;-\;cl\;-\;3n\right)\;\;+\;3s\;+\;2.5p\;+\;0.5k\;-\;o\right]}{M_{n}}$$

where: *c*, *h*, *p*, *s*, *n*, *cl*, *k*, *o*—the amount of individual elements in the macromolecule of biodegradable material; *M*_n_—molecular weight of biodegradable material (g/mol).

## 3. Results and Discussion

### 3.1. Foaming Process

The course of the foaming process depends on the used raw materials [64]. Development of the appropriate composition of the polyol mixture of RPU/PIR foam allows obtaining a product with the desired properties [65]. The blowing agent used in the foaming process has a major influence on the thermal insulation properties of the foams. The Montreal Protocol limits the production and use of ozone-depleting substances. Therefore, CO_2_ and pentane are now widely used as blowing agents [66]. An appropriate suitable catalytic system and surfactant gives a cellular structure that ensures the stability of the performance-usable parameters. Four foams were synthesized according to the formulations presented in Table 2. The basic technological parameters of the foaming of PUFs are processing times. Values of processing times were shown in Table 3. Addition of a bio-fillers (cinnamon, cocoa, or coffee extracts) in the formulation contributed to the elongation of the processing parameters of all RPU/PIR foams. Cream times for obtained foams (C10_0, KZ10_0, EK10_0) were elongated from 8 s for foam W_0 to 10 s for foams with extracts. Free rise times were also increased from 34 s for reference foams to 48 s for C10_0 (foam with 10 wt % of cinnamon extract), to 58 s for KZ10_0, (foam with 10 wt % of coffee extract), and to 45 s for EK_0, (foam with 10 wt % of cocoa extract). String gel times was from 23 s for W_0 to 31 s for EK10_0. Maximum reaction temperatures were increased from 126 °C for reference foam to 165 °C for modified foam (KZ10_0). The process of free rise of the foam and its gelation was disturbed by the addition of extracts. Fillers caused increase in viscosity of the initial premix and thus the free rise time and string gel time increased. Elongation of these times is advantageous when obtaining the molded foams, in which the polyol premix must thoroughly fill the entire mold e.g., in RIM method. 

### 3.2. Organoleptic Assessment of RPU/PIR Foams

**Figure 1:**
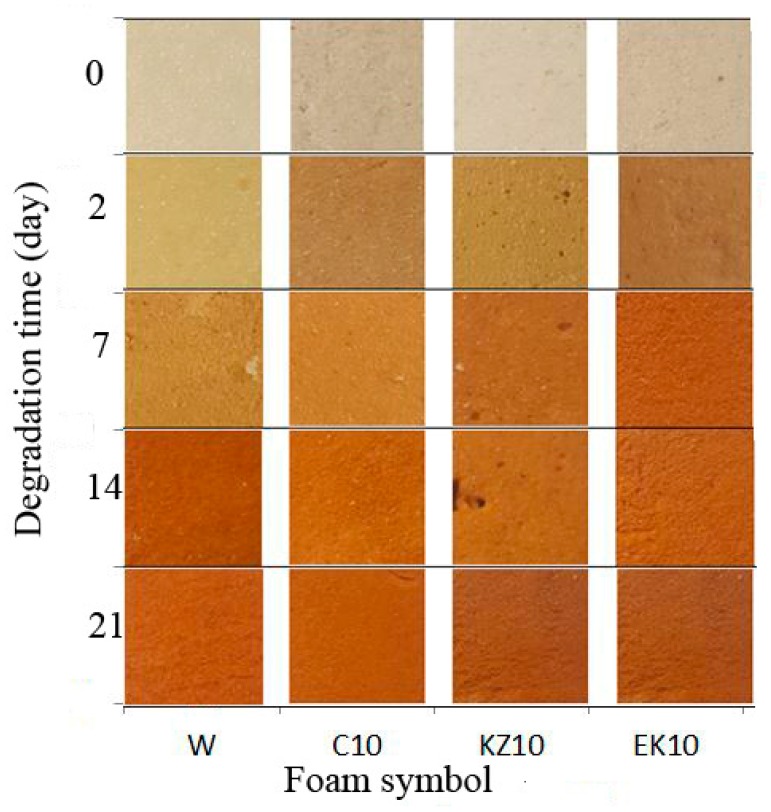
Change in the color of foams after the drying process in the air dryer (2-day) and degradation in the climatic chamber (7, 14, 21-day) and nondegraded foam.

An oraganoleptic analysis was performed comparing the appearance of nondegraded foams with foams aged in an air dryer at 120 °C for 48 h (2 days) and with foams degraded in the climatic chamber (7, 14, 21 days)—Figure 1. The external appearance of the foams and the possible color change were compared. It was determined whether the surface of the foams was crumble or not. Foams after thermostating in the dryer changed their color from light beige-grey (0 days) to grey-green-light brown (2 days) and more red and yellow (7–21 days). Their roughness also increased. The color change to more red and yellow can be caused by thermal-oxidation degradation of the foam surface. This degradation was caused by exposure to UV radiation, humidity, and temperature.

The reason of yellowing of polyurethane surfaces is the formation of colored quinonoimide groups in PU obtained from aromatic MDI [54]. It does not affect the strength properties. Aromatic amines (formed in the reaction of isocyanate with water) are susceptible to oxidation to chromophores form and may affect the color of the foam surfaces [67]. The presence of carbonyl groups caused them to absorb ultraviolet light. They initiated the free radical oxidation process of the polymer which could be done through: Molecules whose excited states behave as initiating radicals (e.g., carbonyl compounds) and molecules that dissociate into free radicals (e.g., peroxides, transition metal salts) [17].

### 3.3. Color of Foam

**Figure 2:**
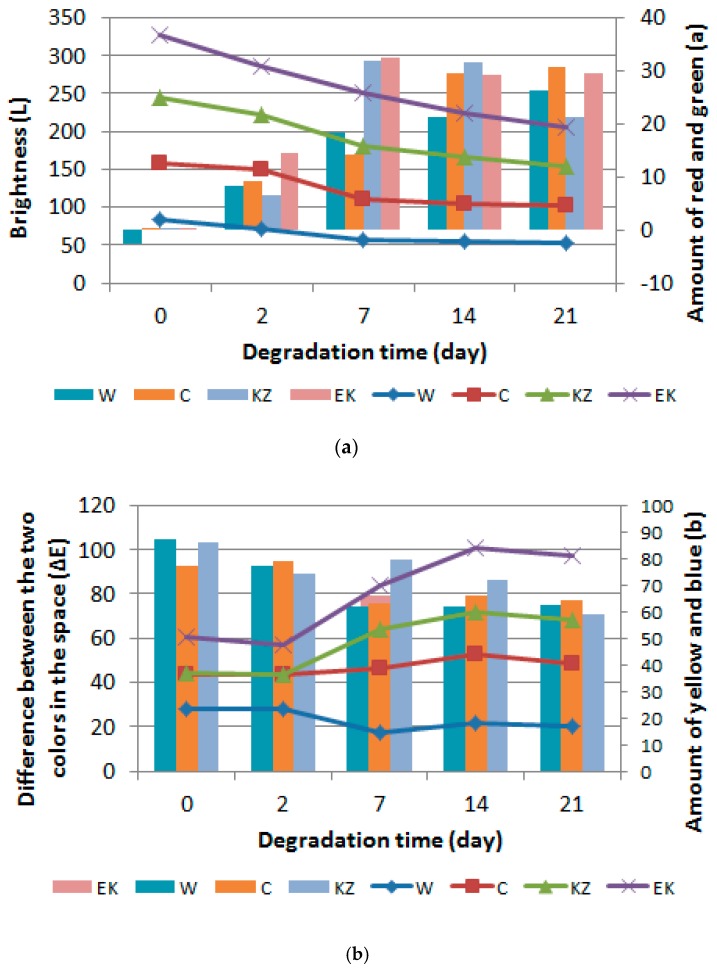
Foam color measurement results of foams after the drying process in the air dryer (2-day) and degradation in the climatic chamber (7, 14, 21-day) and nondegraded foam: (**a**). Change in brightleness-L and amount of red and green -a; (**b**) Change in difference between the two colours in the space -∆E and amount of yellow and blue -b.

Foam color test results were presented in Figure 2a,b. The test results showed a decrease in the brightness of the foams with increasing time of degradation (from 7 to 21 days) in all foams (W, C, KZ, EK), e.g., brightness L was from 86.214 for KZ10_0 foam to 51.432 for KZ10_21. It meant that the samples darken—Figure 2a. An increase of conditioned time also increased the content of red and green (a) and yellow and blue (b) colors. W_0 foam had the largest ∆E parameter (87.461), while the KZ10_21 foam had the lowest (58.951). The value of ∆E decreased with the longer degradation time from 87.461 for W_0 foam to 62.757 for W_21 foam, to 64.231 for C10_21 foam, to 58.951 for KZ10_21 foam, and to 66.930 for EK10_21 foam—Figure 2b.

The fragility and porosity of the surface also increased. The conditions created in the air conditioner caused the crack of thinnest walls of the foam cells and cracked cell skeletons. 

### 3.4. Accelerated Aging Test

Aging of foams was determined by examining the change of linear dimensions (horizontal and vertical), change of geometrical volume, and mass loss. RPU/PIR foams tested by thermostating at 120 °C in a dryer with forced circulation during 48 h caused slight changes of Δ*l*, Δ*V*, and Δ*m* (Table 4). Standards in civil engineering allow for these changes of 3% (for Δ*V*) and 1% (for Δ*l*) [67]. The KZ10_2 and EK10_2 foams had the highest aging resistance because their ∆m values were the lowest and were −0.56 and +0.67, respectively. This meant that the extracts of green coffee and cocoa contained in the foams increased the aging resistance of this foams.

### 3.5. Compressive Strength and Density

**Figure 3:**
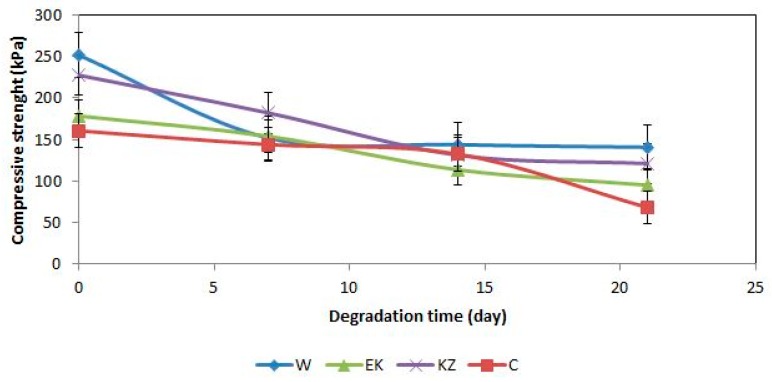
Dependence between compressive strength and degradation time of foams.

The aging time of the foams reduced their compressive strength (Figure 3). Foam W_0 had the highest value of compressive strength (CS) before degradation (251.6 kPa). This foam had the lowest value of CS (W_21 foam, 140.6 kPa) after 21 days of degradation. A similar decrease in this property was observed for the other foams modified by extracts, e.g., for C10_0 foam as 160.5 kPa, for C10_21 foam CS was 68.1 kPa.

The literature gives [60,61] that the assessment of the results of aging tests can be made by specifying the coefficient of variation (CV) in relation to the aging. CV in aging was calculated as a ratio of compressive strength of 0 and 7 series of foams (CV1), 7 and 14 series of foam (CV2), 14 and 21 series of foam (CV3) (Table 5). The choice of this property for the assessment of aging was made due to its importance in the application of polyurethane foams in civil engineering. The CV coefficient of the tested foams was also reduced similarly to the compressive strength of foams, e.g., for the W series from 60.3% (CV1) to 55.9% (CV3), for the C series from 89.7% (CV1) to 42.5% (CV3), for KZ series from 80.0% (CV1) to 53.2% (CV3), and for the EK series from 86.3% (CV1) to 53.3% (CV3).

The apparent density of the foam determines its mechanical properties. Low density of PU materials is economically advantageous and more beneficial for civil engineering [14]. The results of research on foams modified by bio-fillers (Table 5) showed that the apparent density of foams did not significantly change relative to the density of non modified foam and was in rage of 35–42 kg/m^3^ [37,68,69]. 

### 3.6. FTIR Analysis

**Figure 4:**
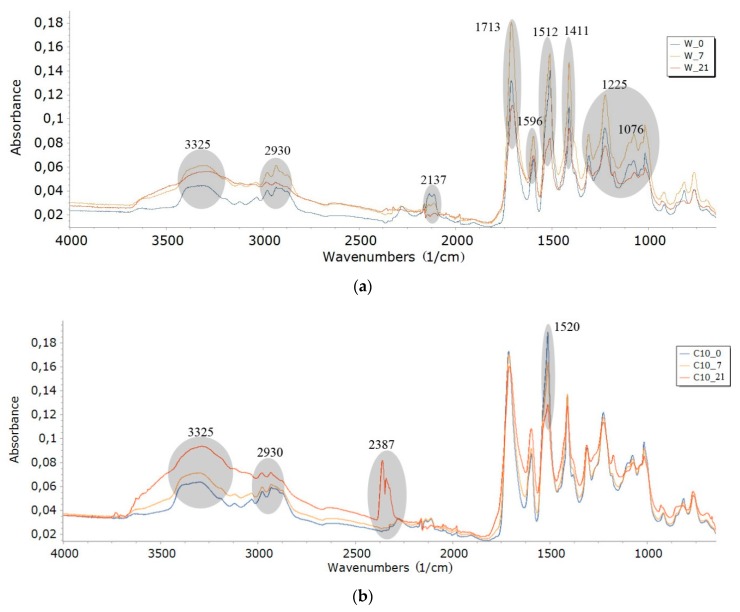

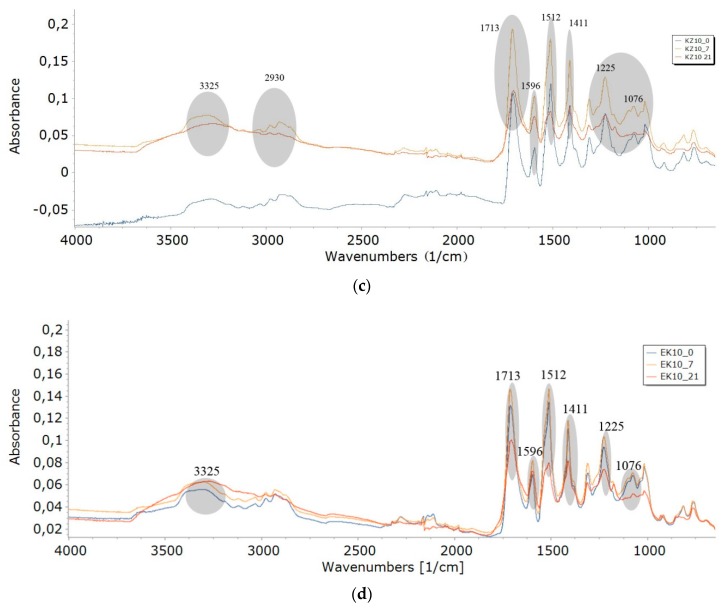
FTIR spectra of: (**a**) W series; (**b**) C series; (**c**) KZ series; and (**d**) EK series.

Photodegradation of polymeric materials is most intensively in the surface layer, up to approximately 10 μm [70]. The surface of nondegraded (0 series of foams) and degraded foams (7, 14, and 21-days series) was scraped and subjected to an infrared spectoscopy analysis. FTIR spectra of degraded foams were compared with the spectra of nondegraded foams (0-days series). Results of this analysis were shown in Figure 4a–d. 

FTIR analysis of selected degraded and nondegraded foams was performed (Figure 4). The bands for which there was a change in intensity at individual wavenumber ranges for degraded foams (7, 14, or 21-days) were compared with the bands of nondegraded foam (0-days). These comprisions were marked with a gray ellipse. The following groups (Table 6) can be highlighted in the structure of all foams on the basis of the FTIR analysis (Figure 4), e.g., N–H, CH, –N=C=O, –N=C=N, C=O in a urethane and isocyanurate ring, also C–O and –C=N in the trimer. These results confirmed the polyurethane-polyisocyanurate foam structure [12,13,56]. 

Susceptibility to degradation depends on the presence of specific chemical groups in the molecule. The easily hydrolysable ester, amide, and urea groups accelerate the polymer decomposition [69,70]. 

An increase in absorbance of some bond bands was observed regardless of the type of foam (W, C, KZ, EK), which indicated structural changes taking place in the foams. The bands increased their intensity with the increased degradation time in the air conditioner chamber. Intensity of wavenumbers increased for the remaining bands (3325, 1713, 1596, 1512, 1411, 1225, 1076 cm^−1^) for W series of foams—Figure 4a. An increase in band intensity was observed in the case of foams with cinnamon extract (C series) at wavenumbers of 2930, 2387, and 2137cm^−1^, and decrease in band intensity for wavenumber of 1520 cm^−1^—Figure 4b. The changes were slight or nonexistent for the other wavenumbers ranges of the C series. The FTIR spectra for foams with green coffee extract (Figure 4c, EKZ series) showed a decrease in the intensity of bands for all 21-day degraded foam (KZ10_21) in comparision with bands for the 7-day degraded foam (KZ10_7)—Figure 3c. The FTIR spectra for foams with cocoa extract (Figure 4d, EK series) showed an increase in the intensity of bands for wavenumbers of all degraded foam (EK10_14, EK10_14, EK10_21) in comparision with bands of nondegraded foam (EK10_0).

It is not exactly known after a given dose of UV radiation, humidity, and temperature over an analyzed period of time, which of these climatic factors had the greatest impact on the increase in absorbance. We do not know whether each individual factor would cause different intensity of absorption bands. The tests were performed in standardized conditions. The dose of UV radiation, humidity, or temperature may be changed in a given time depending on the conditions of use of the foams. It can only be concluded based on the known RPU/PIR properties that UV radiation had the greatest influence on the foam destruction process, because PUs is not resistant to ultraviolet rays [55]. It can be seen from Figure 4 that the addition of cinnamon extract blocked the destruction of some bonds (Figure 4b). Despite the fact that cinnamon contained only 5% of polyphenols, including flavonoids (cocoa and coffee extract had 45% of each), it was the best protection against destruction of bond like: –C=O in urethane bond (1713 cm^−1^), isocyanurate ring (1411 cm^−1^), C=N in trimer (1225 cm^−1^), or C–O (1076 cm^−1^). Research on the antioxidant properties of flavonoids was carried out by Czaplińska et al. [58]. Antioxidant properties of flavonoids were determined by the presence of hydroxyl groups in rings by the isomeria of their location, and the presence of a double bond and a carbonyl group in the heterocyclic ring. The antioxidant properties of flavonoids are determined by the presence of a catechol group in the benzene ring in combination with a hydroxyl group in the C3 position, pyrogallol (trihydroxy) group in the benzene ring, a double bond between C2 and C3 carbon, a carbonyl group in combination with a double bond between carbon atoms (C2 and C3 in the heterocyclic ring), hydroxyl groups at the C5 and C7 positions of the heterocyclic ring.

### 3.7. Differential Scanning Calorimetry

**Figure 5:**
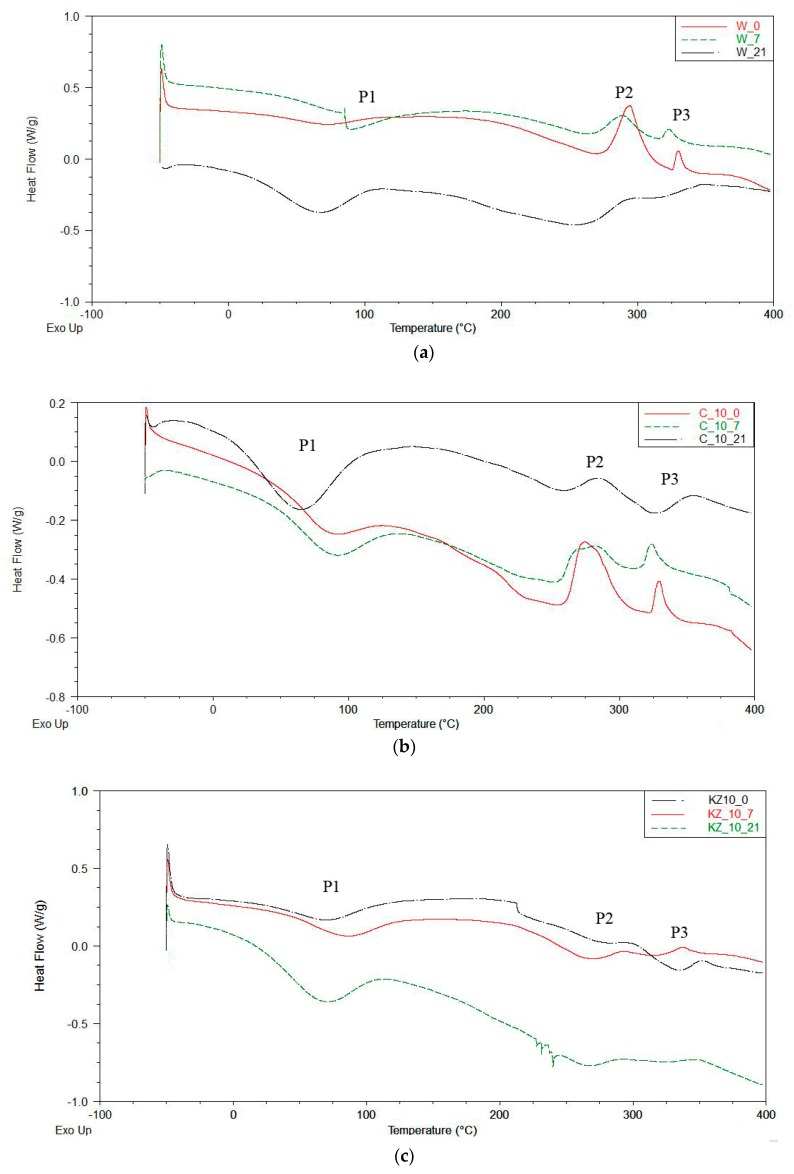

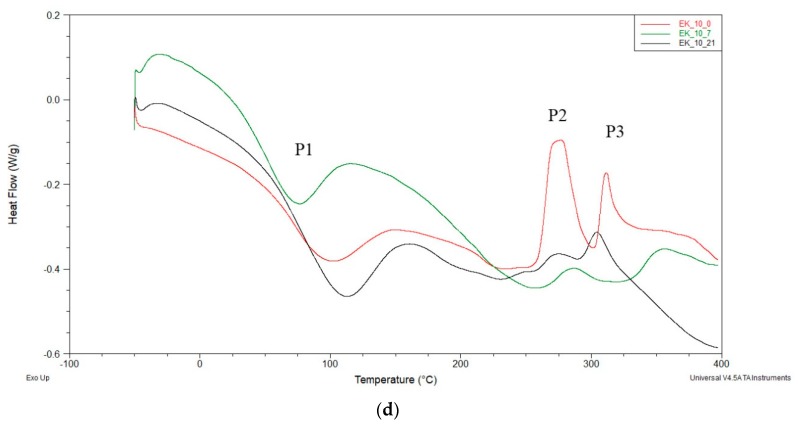
DSC thermograms of: (**a**) Unmodified foam (W series); (**b**) foam modified by cinnamon extract (C series); (**c**) foam modified by green coffee extract (KZ series); (**d**) foam modified by cocoa extract (EK series).

The DSC curves recorded effects, such as: The temperature of the beginning of the thermal effect (*T*_oneset_), the temperature of the end of the thermal effect (*T*_k_), the temperature of the extreme point (*T*_max_), and enthalpy (H) [71,72,73]. The DSC thermograms of cinnamon, green coffe, and cocoa-filled foams (C, KZ, and EK series) had one endothermic peak P1 and two exothermic peaks P2 and P3 (Figure 5a–d). 

It can be noted from the thermograms (Figure 5), that the enthalpy H_1_ in peak P1 increased when degradation time increased (from 0 days to 21 days). H_1_ increased for W series of foams from 25.75 (W_0) to 68.10 J/g (W_21), for C series form 33.38 (C10_0) to 98.32 J/g (C10_21), for KZ series from 19.56 (KZ10_0) to 81.72 J/g (KZ10_21) and for EK series from 47.43 (EK10_0) to 70.11J/g (EK10_21). Detailed values are shown in Table 7. Enthalpy H_1_ was associated with the evaporation of water from foams and with destruction some of filler ingredients. Its increase was caused by the increasing content of the bio-fillers in the foams. Enthalpy H_2_ was different for all foams and it was not observed any correlation between H_2_, degradation time or type of filler. Enthalpy H_3_ increased (three times) in the W and C series of foam and dereased in the KZ series (twice) and in EK series (three times) with decreasing degradation time. Decreasing enthalpy H_3_ probably depended on the composition of the used fillers (Table 1) and products obtained from foam degradation after 21 days in climatic chamber. The highest temperatures at the maximum of individual peaks (*T*_max1_, *T*_max2_ and *T*_max3_) were observed for foams degraded during seven days. A lower temperature *T*_max_ was observed for nondegraded foams and for foams degraded during 21 days regardless of the type of used filler (W, C, KZ, EK). There was not any dependance between the content of the tested extracts and the *T*_oneset_ and *T*_endset_ temperatures. The changes in the peaks (peak P2 and P3) observed in Figure 4 could indicate the breakdown of urethane (with dissociation temperature of 200 °C) and ether bonds (with dissociation temperature of 260 °C) [74,75,76]. Foams subjected to biodegradation in soil could not be tested by DSC because it was not possible to extract them from the soil solution.

### 3.8. RPU/PIR Foams Structure

Properties of RPU/PIR foams are strongly dependent on the cellular structure. Foams structure was analyzed by using an SEM microscope. The structure of foams with cells diameter smaller than 0.25 mm is referred to as a finely pored structure [45]. Cells with a diameter above 0.5 mm are referred to as large. The small-cell structure gives porous materials a more favorable mechanical strength.

The anisotropy coefficent > 1 means the elongation of cells in the vertical direction. The highest strength parameters are characterized by foams with anisotropy coefficient equal to 1 (not elongated neither vertically nor horizontally) [77]. Anisotropy of analyzed foams was about 1 (Table 8). This meant that the processing parameters (Table 2) were long enough to create a spherical shape of cells.

**Figure 6:**
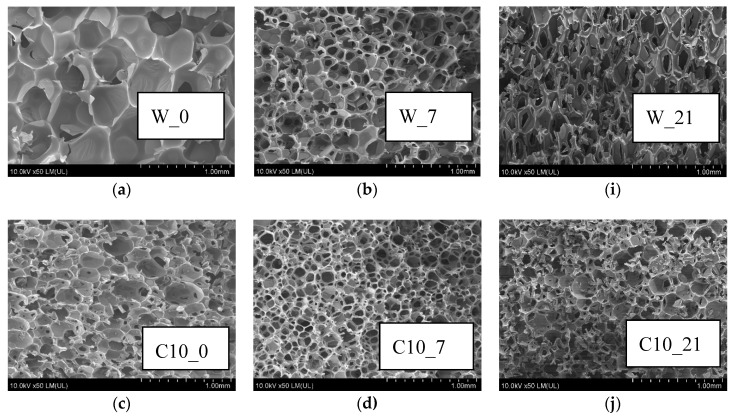

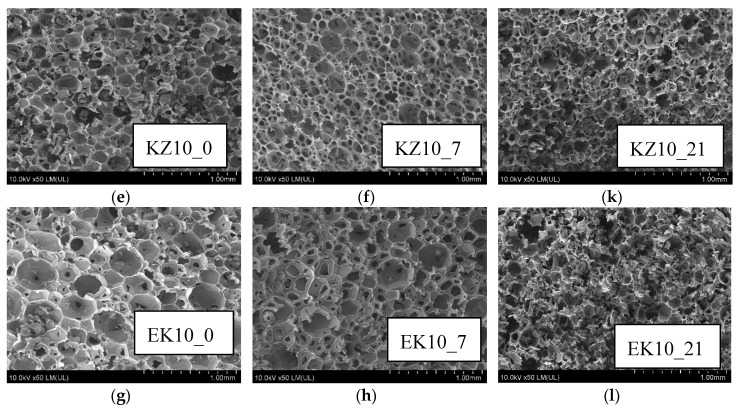
SEM micrographs of foams: (**a**) W_0, (**b**) W_7; (**c**) C10_0; (**d**) C10_7; (**e**) KZ10_0t; (**f**) KZ10_7; (**g**) EK10_0; (**h**) EK10_7; (**i**) W_21; (**j**) C10_21; (**k**) KZ10_21; (**l**) EK10_21.

The micrographs of the obtained foams showed that the cellular structure of the modified (C, KZ, and EK) and unmodified (W) foams was homogeneous (Figure 6a,c,e,g). The cell walls were destroyed during conditioning (Figure 6b,d,f,h) (under given conditions) and additional holes were formed. These additional holes were interpreted in the SEM study and measured by the program as cells (and not as holes created as a result of wall destruction). On a given surface (in this case 1 mm^2^) additional holes were interpreted as smaller cells (not as real cells). In fact, the results in Table 8 relate to measurements of holes created after destruction and not measurements of real cells. Measurement proved that foams conditioning (series 7-days) were destroyed (the thinnest fragments of the walls were broken)—Figure 6b,d,f,h. In this way, a 7.58 cell/mm^2^ was obtained for C10_7 foam, while a 3.88 cell/mm^2^ was obtained for W_0 foam (Table 8). For the 21-day series foams (Figure 5i–l), the number of cells per 1 mm^2^ increased about five times.

Foams containing raw materials of plant origin (Figure 6d,f,h) and reference foam (Figure 6b), which did not contain these fillers, were degraded. Cracking ribs of the cell walls of the foams (Figure 6b,d,f,h) caused the foam surfaces to be rough and brittle [78]. In foam cells degraded for 21 days (Figure 6i–l), not only the walls degraded but skeletons of foam cells cracked too. The most degraded surface had foam containing cocoa extract, subjected to 21 days of climatic factors in the conditioner (EK10_21, Figure 6l).

### 3.9. Biodegradation of RPU/PIR Foams

Biodegradation tests are useful, among others, for determining permissible concentrations of pollutants in soil, sewage, and surface waters and for predicting biodegradability of organic compounds in waters and soils [17]. Biodegradation in soil were conducted by Tosin et al. [79]. They used a method based on measuring the amount of CO_2_ separated from a polymer containing 65% of polyesters and 28% of starch. They examined the biodegradation rate. This is a parameter that is necessary to predict the polymer′s environmental fate. In addition to CO_2_, methane can also be a biodegradable product [80]. Microorganisms can break down organic chemicals that cannot be changed significantly by higher organisms. Aranguren et al. [15] degradation substrates prepared: Soil enriched with composted pine needles with its natural microflora and vermiculite inoculated with a mixed culture capable of degrading phenolic contaminants, as well as anilines include Pseudomonas aeruginosa and Achromobacter Marplatensis. The degradation assessment was based on water sorption (WS) and weight loss (WL) during soil and vermiculite experiments. The WS was slightly higher in soil than in vermiculite after 300 days. The fastest weight loss was observed during the first 60 days.

The susceptibility to biodegradation of foams was assessed. The elemental composition of the foams tested in an OxiTop apparatus was determined (Table 9). The results are given in mass shares. These analyses showed slightly lower values of carbon, hydrogen, oxygen, chlorine, silicon, nitrogen, phosphorus, chlorine, and potassium in the bio-based composite. The content of carbon, nitrogen, phosphorus, potassium, and chlorine in all tested foams decreased in comparison with the W foam. The content of hydrogen in EK10 foam also decreased. Elemental composition analysis is necessary to determine TOD, that is the theoretical amount of oxygen necessary for total mineralization of the tested sample. The big advantage of the modified foams (C10, KZ10, EK10) is a much higher biodegradation degree than in the reference foam (W). This is due to the fact that elements of natural origin (e.g., carbon, hydrogen, nitrogen, oxygen) have been introduced with plant fillers. Literature reports that elements of natural origin are much more easily biodegradable than elements of petrochemical origin [81,82]. This means that they can improve the biodegradability of foams. However, it would be necessary to prepare a suitable biodegradable environment (e.g., soil or compost) to carry out the biodegradation process. 

**Figure 7:**
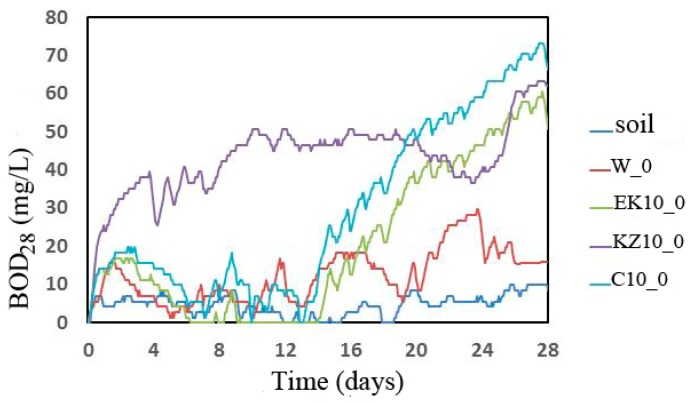
Dependence of BOD changes in time.

The graph of BOD changes during the research of susceptibility to biodegradation (28 days) is shown in Figure 7. 

The BOD_28_ values of analyzed samples, necessary to determine the D_t_ after 28 days in the soil environment, were calculated based on the results of BOD changes of foams (Figure 7) and BOD changes of soil environment (without foams). The TOD values of the appropriate foam were calculated based on the results from Table 10 and Equation 11. The biodegradation degrees (Table 10) were calculated from Equation 10. Research showed that the KZ10_0 foam had the highest values of biochemical oxygen demand, especially up to day 19 of biodegradationc (Figure 7). However, the highest BOD values were achieved by EK10_0 and C10_0 foams after the 19th day of degradation. BOD after 28 days of research of susceptibility to biodegradation reached the highest value for C10_0 (57.7 mg/L). Theoretical calculations of oxygen demand and biodegradation showed that the largest TOD was for W_0 foam (81.27 mg/L). Cinnamon modified foam was characterized by the biggest biodegradation degree D_t_ value (78.65%). D_t_ was the lowest for W foam and was 8.3% and BOD was 7%.

This method did not allow assessing the properties of foam after biodegradation (e.g., DSC or FTIR). It was impossible to separate the foam after the biodegradation test that was in the soil–water solution.

## 4. Conclusions

Four foams were obtained: W—reference foam, C—foam with extracts of cinnamon, KZ—foam with green coffe, EK—foam with cocoa. The results of this research showed improvement of some properties of foams with a bio-filler. The measurement of the color indicated an increase in the red and yellow colors of foams subjected to conditioning (accelerated degradation conditions) and an increase in the color of blue in thermostated foams (for 48 h in an exhaust dryer). Studies have shown that the addition of coffee and cocoa extract increased the aging resistance of foams (∆*m* was less than 1%) in comprison with foam without a filler (over 3% for reference foam W_2) and foam with cinnamon extract (over 4% C10_2 foam). The compressive strength (CS) values for degraded foams (maximum degradation time 21 days) decreased, compared to CS for nondegraded foams about 111 kPa for W foam, about 98 kPa for C foam, about 107 kPa for KZ foam, and about 83 kPa for EK foam. It was found on the basis of SEM analysis that foams modified with plant-based fillers and reference foam were degraded under the influence of UV radiation, moisture, and temperature in a similar way. The larger number of cells in foams degraded per 1 mm^2^ was caused by the cracking of the thinnest cell walls, which created additional cells (holes) on the foam surface. The SEM study showed the spherical shape of the foam cells. Biodegradation tests have shown that foams modified by plant-based fillers (cinnamon, green coffee, and cocoa extracts) were more susceptible to biodegradation than the reference foam. They were characterized by about seven-times higher BOD values after 28 days of biodegradation than the reference foam. They have less than 10 mg/L lower TOD values and 7–9 times higher *D*_t_ values.

The flammability, thermal, and other performance properties of the tested foams will be the subject of the next article.

## Figures and Tables

**Table 1:** Extracts composition: C—cinnamon, KZ—green coffee, EK—cocoa.

Compound	Content in C10 Foam	Content in KZ10 Foam	Content in EK10 Foam
Polyfenols (e.g., chlorogenic acid, flavonoids, phenol acids)	5.0%	45.0%	5.0%
Minerals	<2.0 ppm	4.4%	<0.3 ppm
Carrier to active substances (e.g., cellulose, lignin, polysaccharides)	about 94.9%	about 50.6%	about 94.9%

**Table 2:** Formulation of RPU/PIR foams.

Foam	Filler (wt %)		
	Cinnamon	Green Coffee	Cocoa
W_0	0	0	0
C10_0	10	0	0
KZ10_0	0	10	0
EK10_0	0	0	10

**Table 3:** Processing times of RPU/PIR foams.

Foam	Cream Time (s)	Free Rise Time (s)	String Gel Time (s)	Tack Free Time (s)	*T*_max_ (°C)
W_0	8	34	23	21	126
C10_0	10	48	30	25	146
KZ10_0	10	58	29	23	165
EK10_0	10	45	31	24	152

**Table 4:** Results of foam aging measurement in an air dryer (120 °C, 48 h).

Foam	∆*l* (%)	∆*V* (%)	∆*m* (%)
W_2	+0.39 ± 0.01	−1.77 ± 0.01	+3.27 ± 0.01
C10_2	+0.80 ± 0.01	−2.94 ± 0.01	+4.35 ± 0.01
KZ10_2	−0.12 ± 0.01	+0.08 ± 0.01	−0.56 ± 0.01
EK10_2	+0.20 ± 0.01	−2.39 ± 0.01	+0.67 ± 0.01

**Table 5:** Density (d), compressive strength (W), and compressive strength ratio (CV): W_0_—measured before conditioning, W_7_—mesaured after 7-days conditioning, W_14_—mesaured after 14-days conditioning, W_21_—mesaured after 21-day conditioning.

Foam	d (kg/m^3^)	W_0_ (kPa)	W_7_ (kPa)	W_14_ (kPa)	W_21_ (kPa)	CV1 (%)	CV2 (%)	CV3 (%)
W_0	39.7 ± 0.1	251.6 ± 1.4	151.8 ± 1.3	143.8 ± 1.3	140.6 ± 1.3	60.3 ± 0.5	57.2 ± 0.4	55.9 ± 0.4
C10_0	35.7 ± 0.1	160.4 ± 1.3	143.9 ± 1.3	132.5 ± 1.3	68.1 ± 1.1	89.7 ± 0.5	82.6 ± 0.4	42.5 ± 0.3
KZ10_0	42.9 ± 0.1	227.3 ± 1.4	181.9 ± 1.4	131.1 ± 1.2	120.9 ± 1.2	80.0 ± 0.5	57.7 ± 0.4	53.2 ± 0.4
EK10_0	35.7 ± 0.1	178.3 ± 1.4	153.9 ± 1.3	113.5 ± 1.3	95.1 ± 1.1	86.3 ± 0.5	63.7 ± 0.4	53.3 ± 0.4

**Table 6:** Results of FTIR analysis.

Band (cm^−1^)	Bond
3325	N–H
2930	C–H
2276	–N=C=O
2137	–N=C=N–
1713	–C=O in urethane bond
1596	N–H
1512	N–H
1411	Isocyanurate ring
1225	C=N in trimer
1076	C–O

**Table 7:** Values of thermal transformations of W, C, KZ, EK foams series.

Foam	Peak P1				Peak P2				Peak P3			
	*T*_onset_, (°C)	*T*_max1_ (°C)	*T*_k1_ (°C)	H_1_ (J/g)	*T*_onset2_ (°C)	*T*_max2_ (°C)	*T*_k2_ (°C)	H_2_ (J/g)	*T*_onset3_ (°C)	*T*_max3_ (°C)	*T*_k3_ (°C)	H_3_ (J/g)
**W_0**	21.7	71.8	146.6	25.75	278.6	294.8	325.3	44.86	326.3	330.4	347.3	5.08
**W_7**	54.7	91.3	179.8	41.76	271.5	289.0	314.4	18.95	318.2	323.6	342.2	4.73
**W_21**	14.6	64.6	106.2	68.10	166.1	253.2	294.6	87.41	322.4	347.8	393.3	16.68
**C10_0**	46.7	86.4	139.4	33.38	261.9	274.5	319.1	35.60	324.0	329.8	352.7	6.06
**C10_7**	39.7	93.9	133.9	47.54	259.7	285.3	313.0	13.83	331.2	337.5	358.8	5.24
**C10_21**	10.2	63.2	146.6	98.32	263.5	286.5	323.8	14.32	332.6	353.9	396.1	12.05
**KZ10_0**	15.9	68.0	96.0	19.56	213.0	247.7	297.8	18.40	304.3	331.9	352.0	17.17
**KZ10_7**	21.3	84.8	139.4	44.23	200.7	263.2	291.0	22.12	295.4	319.1	337.2	6.02
**KZ10_21**	17.3	66.7	109.8	81.72	231.1	240.1	292.2	24.02	328.9	351.1	385.9	10.81
**EK10_0**	50.3	96.6	150.6	47.43	260.6	274.6	299.3	36.89	304.3	311.6	363.7	17.50
**EK10_14**	23.4	70.3	114.6	79.78	163.6	249.4	291.8	63.97	309.7	348.5	394.8	19.86
**EK10_21**	51.9	107.5	153.3	70.11	262.3	272.8	289.1	1.97	293.5	304.5	317.4	5.81

**Table 8:** Results of SEM micrographs analysis.

Foam Symbol	Cell/Hole Height (µm)	Cell/Hole Width (µm)	Anisotrophy Coefficient (-)	Cell/Hole Surface Area (mm^2^)	Content of Cell/Hole Per Area Unit (cell/mm^2^)
W_0	606.6 ± 2.2	540.7 ± 0.2	1.12 ± 0.01	0.258 ± 0.001	3.88 ± 0.01
W_7	367.7 ± 1.1	340.9 ± 0.1	1.08 ± 0.01	0.132 ± 0.001	7.58 ± 0.01
W21	267.8 ± 1.1	219.6 ± 0.1	1.22 ± 0.01	0.046 ± 0.001	21.74 ± 0.01
C10_0	346.7 ± 1.1	324.9 ± 0.1	1.11 ± 0.01	0.177 ± 0.001	5.65 ± 0.01
C10_7	311.4 ± 1.1	299.0 ± 0.1	1.04 ± 0.01	0.146 ± 0.001	6.85 ± 0.01
C10_21	218.0 ± 1.1	226.8 ± 0.1	0.96 ± 0.01	0.038 ± 0.001	26.32 ± 0.01
KZ10_0	303.4 ± 1.1	279.2 ± 0.1	1.09 ± 0.01	0.133 ± 0.001	7.52 ± 0.01
KZ10_7	289.1 ± 1.1	277.5 ± 0.1	1.04 ± 0.01	0.126 ± 0.001	7.94 ± 0.01
KZ10_21	220.7 ± 1.1	222.3 ± 0.1	0.99 ± 0.01	0.384 ± 0.001	26.0 ± 0.01
EK10_0	356.4 ± 1.1	351.2 ± 0.1	1.01 ± 0.01	0.197 ± 0.001	5.08 ± 0.01
EK10_7	346.7 ± 1.1	344.9 ± 0.1	1.00 ± 0.01	0.188 ± 0.001	5.32 ± 0.01
EK_21	208.7 ± 1.1	193.3 ± 0.1	1.08 ± 0.01	0.032 ± 0.001	31.25 ± 0.01

**Table 9:** Mass shares of individual elements in analyzed foams.

Foam	C	H	O	Si	N	P	Cl	K
W_0	0.627 ± 0.001	0.058 ± 0.001	0.183 ± 0.001	0.005 ± 0.001	0.067 ± 0.001	0.013 ± 0.001	0.045 ± 0.001	0.003 ± 0.001
C10_0	0.610 ± 0.001	0.058 ± 0.001	0.209 ± 0.001	0.005 ± 0.001	0.062 ± 0.001	0.012 ± 0.001	0.041 ± 0.001	0.002 ± 0.001
KZ10_0	0.610 ± 0.001	0.058 ± 0.001	0.209 ± 0.001	0.005 ± 0.001	0.062 ± 0.001	0.012 ± 0.001	0.041 ± 0.001	0.002 ± 0.001
EK10_0	0.622 ± 0.001	0.057 ± 0.001	0.199 ± 0.001	0.005 ± 0.001	0.062 ± 0.001	0.012 ± 0.001	0.041 ± 0.001	0.002 ± 0.001

**Table 10:** Results of biodegradability of analyzed foams.

Foam	Sample Weight (g)	BOD_28_ (mg/L)	TOD (mg/L)	*D*_t_ (%)
W_0	0.203 ± 0.001	7.0 ± 0.1	81.27 ± 0.01	8.30 ± 0.01
C10_0	0.211 ± 0.001	57.7 ± 0.1	73.36 ± 0.01	78.65 ± 0.01
KZ10_0	0.207 ± 0.001	53.5 ± 0.1	76.26 ± 0.01	70.16 ± 0.01
EK10_0	0.223 ± 0.001	42.2 ± 0.1	71.77 ± 0.01	58.80 ± 0.01
